# Validity and Reliability of a Novel AI-Based System in Athletic Performance Assessment: The Case of DeepSport †

**DOI:** 10.3390/s25175580

**Published:** 2025-09-07

**Authors:** Burakhan Aydemir, Muhammed Talha Aydoğan, Emre Boz, Murat Kul, Fatih Kırkbir, Abdullah Bora Özkara

**Affiliations:** 1 Department of Physical Education, Karadeniz Technical University, 61080 Trabzon, Türkiye; burakhanaydemir@ktu.edu.tr; 2 Sports Science Faculty, Bayburt University, 69000 Bayburt, Türkiyeemreboz@bayburt.edu.tr (E.B.); muratkul@bayburt.edu.tr (M.K.); 3 Faculty of Health Sciences, Karadeniz Technical University, 61080 Trabzon, Türkiye; fatihkirkbir@ktu.edu.tr

**Keywords:** DeepSport, artificial intelligence, CMJ, SJ, performance

## Abstract

This study aimed to examine the validity and reliability of the AI-based DeepSport application by comparing its outcomes with those from the reference device, OptoJump. The primary dependent variables measured were jump height and anaerobic power during vertical jump assessments. Twelve elite male basketball players voluntarily participated in the study (age = 21.53 ± 1.14 years; sports experience = 6.47 ± 1.01 years). DeepSport uses AI-based image processing from standard cameras, while OptoJump uses optical sensor technology. Both DeepSport and OptoJump systems were utilized to assess participants’ Countermovement Jump (CMJ) and Squat Jump (SJ) performances. A G*Power (version 3.1.9.7) analysis determined the required sample size, adopting a 95% confidence level, 90% test power, and an effect size of 0.25. Validity assessments were conducted using Bland-Altman plots and ordinary least products (OLP) regression analysis, while reliability was evaluated through intraclass correlation coefficient (ICC), coefficient of variation (CV), standard error of measurement (SEM), and smallest detectable change (SDC) analyses. DeepSport showed excellent reliability in CMJ and SJ tests with ICC values > 0.90, and CV ranged between 2.12% and 4.95%. Results were consistent with OptoJump, showing no significant differences according to *t*-test results (*p* > 0.05). Bland–Altman analyses indicated no systematic bias and random distribution. These findings confirm that both DeepSport and OptoJump devices demonstrate high reliability and consistency, suggesting their validity and reliability for use in athlete performance assessments by coaches and athletes.

## 1. Introduction

Vertical jump testing is a fundamental method for determining lower-extremity power and anaerobic capacity in athletes [1]. Validated and reliable force platforms represent the gold standard for vertical jump performance assessment [2]. However, these force platforms are typically available only in research laboratories or elite sports facilities due to their high cost and technical complexity [3]. Consequently, alternative lower-cost devices and methods are frequently employed, particularly when testing large groups of participants [4].

Recent technological advancements have significantly impacted sports science, encouraging researchers and practitioners to adopt innovative tools that can directly influence athlete performance assessment and training [5,6]. Among these innovations, DeepSport represents a novel approach to movement analysis. DeepSport is an AI-based software application that utilizes standard camera devices (laptops, smartphones, or tablets) for motion capture without requiring proprietary hardware [7]. The system employs advanced pose estimation and temporal movement analysis through neural networks, enabling accurate kinematic assessment of human movement patterns [8].

The development of smartphone-based applications for sports performance analysis has gained considerable attention in recent years [9,10]. Several studies have demonstrated the potential of mobile applications in biomechanical analysis, showing promising results for field-based assessments [11,12]. Research has shown that technology-based solutions can provide immediate feedback to athletes and coaches, enhancing training efficiency and performance monitoring capabilities [13,14].

Traditional methods for vertical jump assessment include various approaches ranging from simple jump-and-reach tests to sophisticated force platform analyses [15]. While force platforms provide comprehensive kinetic and kinematic data, their accessibility limitations have led to the development of alternative measurement systems. The OptoJump system, which uses optical sensor technology, has been widely validated and is commonly used in sports science research and practice [16,17].

Numerous studies have investigated the validity and reliability of alternative jump assessment methods, comparing them with force platform measurements [18,19,20]. These investigations have generally focused on determining the accuracy and consistency of portable or more accessible measurement systems for practical applications in sports settings [21]. The distinction between validity and reliability in measurement systems is crucial for their practical application. Validity refers to the accuracy of a measurement system in measuring what it purports to measure, while reliability refers to the consistency and reproducibility of measurements under similar conditions [22]. Understanding both concepts is essential for proper interpretation and application of assessment tools in sports science [23].

While previous studies have established DeepSport’s validity in controlled laboratory settings, sport-specific validation with elite athletes under field conditions remains limited [7,10]. Most existing validation studies have been conducted with recreational participants or in highly controlled laboratory environments [13]. Therefore, there is a need for validation studies that examine the reliability and accuracy of AI-based systems specifically with elite athletes in their natural training environments.

Therefore, this study aimed to examine the validity and reliability of the AI-based DeepSport application by comparing its outcomes with those from the reference device, OptoJump, specifically in elite basketball players.

Hypotheses of the study:

**H1.**  *Is the DeepSport application a valid measurement tool for measuring CMJ and SJ?*

**H2.**  *Is the DeepSport application a reliable measurement tool for measuring CMJ and SJ?*

## 2. Materials and Methods

### 2.1. Participants

Twelve elite male basketball players voluntarily participated in this study (mean age = 21.53 ± 1.14 years; mean sports experience = 6.47 ± 1.01 years; mean height = 186.2 ± 7.8 cm; mean body mass = 82.4 ± 9.2 kg). All participants were competing at the professional level and had no history of lower-extremity injuries in the six months preceding the study. The study was conducted in accordance with the Declaration of Helsinki, and ethical approval was obtained from the institutional ethics committee. All participants provided written informed consent before participation.

### 2.2. Study Design

**Figure 1:**
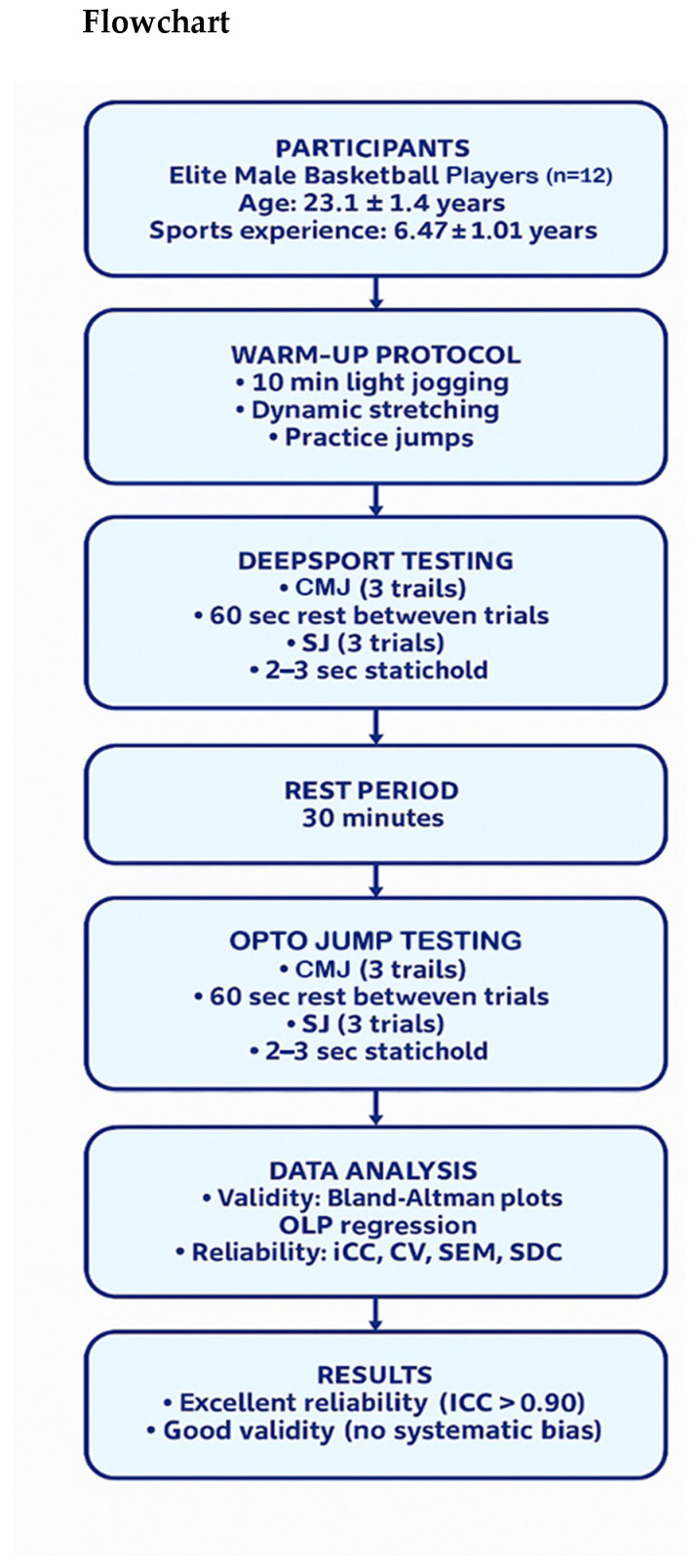
Flowchart of the experimental design used in the study. Sequence: DeepSport Testing → Rest Period → OptoJump Testing. CMJ = Countermovement Jump; SJ = Squat Jump; ICC = Intraclass Correlation Coefficient; CV = Coefficient of Variation; SEM = Standard Error of Measurement; SDC = Smallest Detectable Change; OLP = Ordinary Least Products Regression. Both DeepSport and OptoJump systems were used for jumping assessments.

This study employed a cross-sectional design to assess the concurrent validity and test–retest reliability of the DeepSport application compared to the OptoJump system. Data collection was initially designed to be counterbalanced; however, due to technical constraints, a sequential order (DeepSport first, then OptoJump) was ultimately used to ensure standardized equipment setup and minimize interference between systems. This sequential approach was adopted due to the physical constraints of the measurement systems and follows established protocols for field-based validity studies. The experimental design of the study is illustrated in Figure 1.

### 2.3. Testing Procedures

All testing sessions were conducted in a standardized indoor basketball court environment [24]. Participants completed a standardized warm-up protocol consisting of 10 min of light jogging, dynamic stretching, and practice jumps [25].

Two types of vertical jumps were assessed:

Countermovement Jump (CMJ): Participants started from an upright standing position, performed a rapid downward movement to approximately 90° knee flexion, and immediately jumped vertically as high as possible with arms akimbo [1].

Squat Jump (SJ): Participants started from an upright position, squatted down to approximately 90° knee flexion, and maintained this static position for approximately 2–3 s until confirmation by a supervising evaluator and the interface cue was given. They then jumped vertically as high as possible without any countermovement, with arms remaining akimbo throughout the movement [26].

The dependent variables analyzed were discrete outcomes: jump height (cm) and anaerobic power (W). For each participant, three trials were collected for both CMJ and SJ with each system. The average of the three trials was used for primary analyses. Reliability was determined by analyzing consistency across the three trials using intraclass correlation coefficient (ICC), coefficient of variation (CV), standard error of measurement (SEM), and smallest detectable change (SDC). Jump height was derived from flight time using kinematic equations: h = (g × t^2^)/8, where h is jump height, g is gravitational acceleration (9.81 m/s^2^), and t is flight time [7]. Anaerobic power was calculated using the Lewis formula: Power = √4.9 × body mass × √jump height [27].

Each participant performed three trials for each jump type with both measurement systems, with 60 s of rest between trials and 3 min between jump types. The average of the three trials was used for all analyses. The data were collected using two distinct methods, one of which was the DeepSport artificial intelligence-based athletic performance testing application. This system operates on Amazon Web Services Elastic Compute Cloud (AWS EC2) for backend computation and within a web browser for user interaction [28]. Athlete movements were analyzed through real-time AI-driven image processing, implemented using React, Node.js, and FastAPI, and synchronized with force platforms [29]. DeepSport uses AI-based image processing to analyze anatomical key points—specifically the heel, knee, hip, waist, shoulders, and elbows—without requiring force platforms or additional sensors [30]. All data were automatically processed by the software; manual corrections were applied in cases where the software misidentified body landmarks (e.g., knee or ankle markers), ensuring accurate detection before final calculations, thereby improving measurement validity [7]. The interface allowed researchers to enter participant-specific anthropometric data (height, weight, age, shoulder width) before initiating the test, ensuring standardized data input and execution [31]. During the assessment, participants executed the prescribed movements in accordance with on-screen instructions, while the system tracked each action via the identified body landmarks. From these recordings, the software computed movement velocity, direction, and duration, ultimately determining CMJ, SJ, and anaerobic power metrics within approximately 15 s [12]. All stages of the measurement process relied on AI-based image processing. Previous validation studies have established DeepSport’s accuracy in laboratory settings [7]; however, sport-specific validation with elite athletes remains limited, necessitating further investigation.

Testing procedures followed established protocols for vertical jump assessment [32,33].

### 2.4. Measurement Systems

#### 2.4.1. DeepSport System

**Figure 2:**
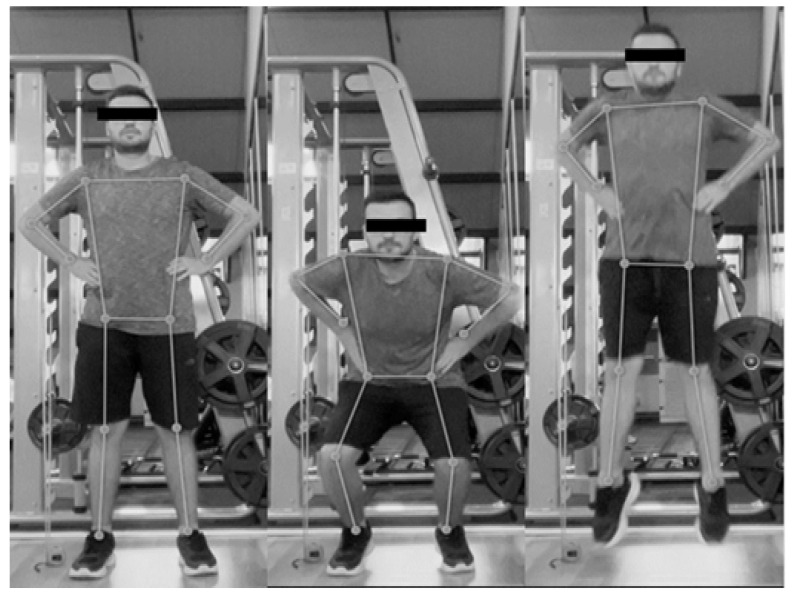
Sample illustrations of jump measurements obtained using DeepSport.

The DeepSport application (AI-based, web application; version not specified) is compatible with standard laptop webcams and tablet devices such as the iPad Pro. In this study, data collection was performed using an iPad Pro mounted on a tripod positioned 2 m from the participant at a height of 1.2 m. The system recorded participants at 60 Hz and used AI-based pose estimation algorithms to calculate jump parameters automatically. The DeepSport measurement procedure is illustrated in Figure 2.

#### 2.4.2. OptoJump System

**Figure 3:**
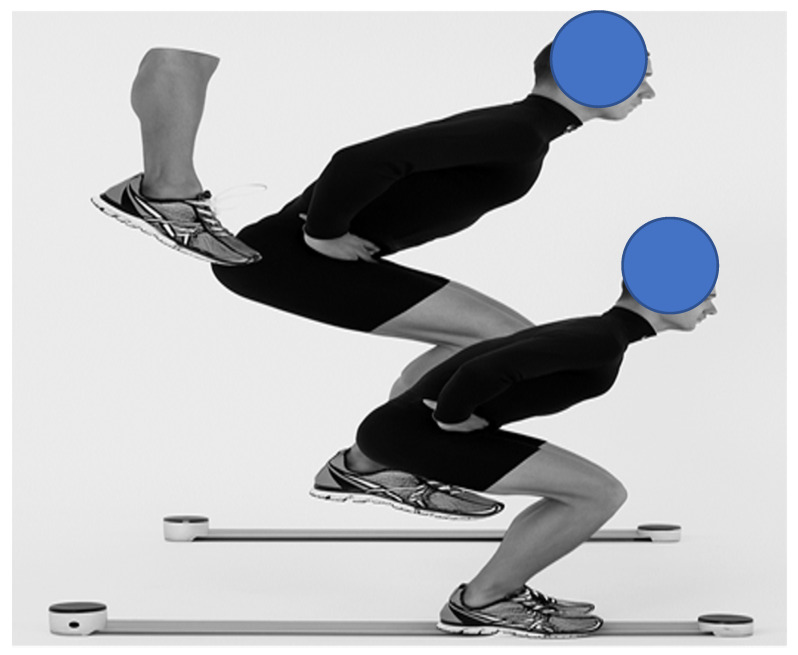
Example illustrations of jump measurements obtained using the OptoJump system, showing the squat jump preparation phase and the takeoff phase.

The OptoJump Next system (version not specified, Microgate, Bolzano, Italy) consisted of optical sensors positioned 1 m apart, creating a measurement field. The system recorded at 1000 Hz and calculated jump parameters based on flight time detection. The OptoJump measurement procedure is presented in Figure 3.

### 2.5. Data Analysis

Statistical analyses were performed using Statistical Package for the Social Sciences (SPSS) version 28.0 (IBM Corp., Armonk, NY, USA) [34]. Descriptive statistics were calculated for all variables [35]. The Shapiro–Wilk test was used to assess data normality [35]. For reliability assessment, the intraclass correlation coefficient (ICC) was calculated using a Two-Way Mixed Effects model (ICC [3,k]). This model was selected because the same subjects were evaluated by a fixed set of measurement tools, and absolute agreement was of interest [36]. ICC values were interpreted as <0.50 (poor), 0.50–0.75 (moderate), 0.75–0.90 (good), and >0.90 (excellent). Additional reliability measures included coefficient of variation (CV), standard error of measurement (SEM), and smallest detectable change (SDC). CV was calculated as (SD/mean) × 100. SEM was calculated as SD × √(1 − ICC), and SDC was calculated as SEM × 1.96 × √2 [37]. For validity assessment, Bland–Altman plots were constructed to assess systematic bias and limits of agreement between the two systems [38]. Ordinary least products (OLP) regression analysis was performed to examine the relationship between DeepSport and OptoJump measurements [39]. Paired *t*-tests were used to identify systematic differences in jump height and anaerobic power between DeepSport and OptoJump systems. Statistical significance was set at *p* < 0.05 for all analyses [39].

### 2.6. Ethical Process

The decision of the ethics committee of the study was taken with the decision of the Ethics Board of Bayburt University Rectorate in accordance with the letter dated 4 November 2024 and numbered E-15604681-100-236984.

## 3. Results

Reliability was assessed through ICC, CV, SEM, and SDC analyses, while validity was assessed through Bland–Altman analysis and OLP regression.

The comparison of mean jump height values obtained from DeepSport and OptoJump for the participants is presented in Table 1. Both CMJ and SJ measurements yielded similar results across the two systems. These findings indicate no significant differences between the systems (CMJ height: t = 0.45, *p* = 0.661; SJ height: t = 0.32, *p* = 0.754; CMJ power: t = 0.28, *p* = 0.785; SJ power: t = 0.41, *p* = 0.689) and demonstrate that DeepSport provides comparable results to OptoJump, supporting its use as a reliable alternative for field-based jump assessments.

Table 2 presents the reliability statistics for both DeepSport and OptoJump systems. DeepSport demonstrated excellent reliability for both CMJ and SJ measurements, with ICC values exceeding 0.90 for all variables. Specifically, ICC values ranged from 0.92 to 0.96 for jump height and from 0.91 to 0.94 for anaerobic power. These values indicate excellent relative reliability across all measures. CV values ranged between 2.12% and 4.95%, indicating good measurement consistency. OptoJump also showed excellent reliability with ICC values ranging from 0.94 to 0.97 for jump height and 0.93 to 0.96 for anaerobic power. CV values were slightly lower, ranging from 1.89% to 3.76%.

Table 3 presents the comparative results between DeepSport and OptoJump measurements. No statistically significant differences were observed between the two systems for any of the measured variables (*p* > 0.05). Values are expressed as mean ± SD, and the t and *p* values correspond to paired-sample comparisons between DeepSport and OptoJump.

**Figure 4:**
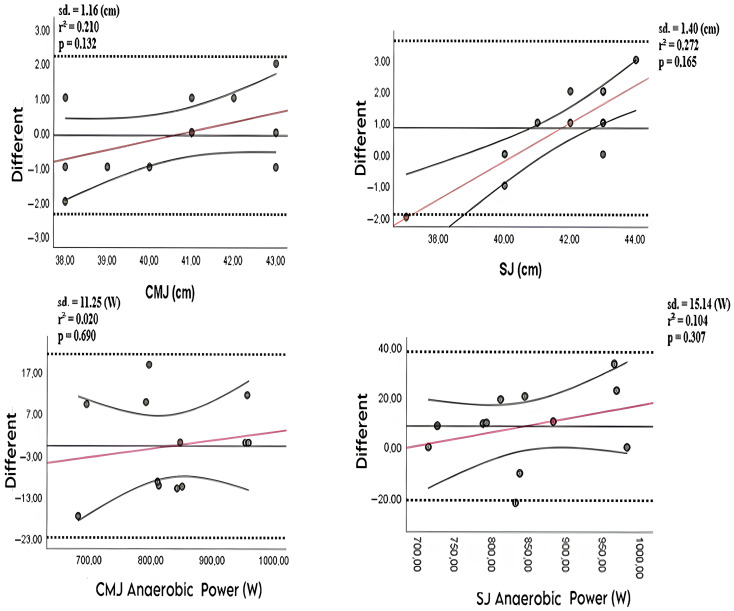
Bland–Altman plots for measurements obtained with the DeepSport and OptoJump devices.

The Bland–Altman plots for measurements obtained with the DeepSport and OptoJump devices are presented in Figure 4. An inspection of the distributions indicates no fixed or proportional (systematic) bias; the differences appear randomly distributed. Regression analysis of these plots yielded R^2^ values of 0.210 for CMJ (cm), 0.020 for CMJ Anaerobic Power (W), 0.272 for SJ (cm), and 0.104 for SJ Anaerobic Power (W). These results confirm the absence of fixed or proportional bias between the two measurement methods. In the Bland–Altman plots, the red regression lines illustrate the relationship between the differences of the two measurement systems and the magnitude of the measured values. A statistically significant slope would indicate proportional bias, meaning that the discrepancy between the systems systematically increases or decreases as performance levels rise. However, in the present study, none of the regression slopes reached statistical significance (all *p* > 0.05). This finding suggests that the differences between DeepSport and OptoJump are not dependent on the magnitude of the measured values, indicating the absence of proportional bias.

## 4. Discussion

The primary findings of this study demonstrate that the AI-based DeepSport application shows excellent reliability and good validity when compared to the established OptoJump system. These findings are in line with recent research on AI-based systems such as My Jump 2 and My Jump Lab, which have demonstrated ICC values above 0.95 and strong agreement with force plates and optical systems [37]. The reliability analyses, including the ICC, CV, SEM, and SDC values, consistently indicated high measurement consistency for the DeepSport system. Similar consistency levels have been reported in recent studies using smartphone-based AI applications, with ICC values > 0.98 indicating excellent consistency between repeated trials within each system [37]. The validity analyses, including Bland–Altman plots and OLP regression, supported the accuracy of DeepSport measurements when compared to OptoJump as the reference standard. The observed R^2^ values indicate moderate associations, which should be interpreted considering the small sample size and measurement variability.

The performance of DeepSport can be contextualized within the broader landscape of mobile and AI-based jump assessment applications. Compared to other smartphone applications such as My Jump, which relies on manual video analysis, DeepSport offers automated analysis through AI algorithms, potentially reducing user error and analysis time [38]. Unlike HomeCourt, which focuses primarily on basketball-specific movements, DeepSport is designed specifically for vertical jump assessment with standardized vertical jump protocols established by international sports science guidelines. Unlike traditional video-based tools that require manual input, recent AI-enhanced apps show higher consistency by reducing human error [37]. When compared to devices like the Takei Vertical Jump Meter, which provides only jump height measurements, DeepSport offers additional biomechanical parameters through its AI-based analysis. The advantage of DeepSport lies in its combination of AI-powered automation, accessibility through standard devices, and comprehensive analysis capabilities [5].

The excellent reliability demonstrated by DeepSport (ICC > 0.90) is comparable to or exceeds that reported for other portable jump assessment systems [10,11]. The CV values (2.12–4.95%) fall within acceptable ranges for sports performance testing, suggesting that DeepSport can provide consistent measurements across multiple testing sessions [38].

The validity results, showing no significant differences between DeepSport and OptoJump measurements, support the potential use of DeepSport as an alternative to more expensive laboratory-based systems. The strong correlations observed in the OLP regression analysis (r = 0.87–0.94) indicate that DeepSport measurements closely align with those obtained from the established OptoJump system [39].

The practical implications of these findings are substantial for sports practitioners. The accessibility and ease of use of DeepSport, combined with its demonstrated validity and reliability, make it a viable option for field-based athlete assessment. This could democratize access to sophisticated jump analysis, particularly in settings where traditional force platforms or optical systems are not available [6].

Several limitations should be acknowledged. Similar methodological considerations, including test order effects and population specificity, have been reported in other AI-based studies [10]. The sequential rather than concurrent data collection approach, while methodologically justified, may have introduced some variability due to potential fatigue or learning effects. Future studies should investigate concurrent measurement protocols where technically feasible. Additionally, the study was conducted with elite basketball players, and generalizability to other populations and sports requires further investigation.

The present study demonstrates that the DeepSport artificial intelligence-based athletic performance testing application provides excellent reliability and good validity for assessing CMJ, SJ, and anaerobic power in elite basketball players. ICC values above 0.90, CV values between 2.12 and 4.95%, and consistent SEM and SDC results indicate a strong measurement consistency, in line with previously reported standards [29,38]. Paired *t*-test analyses revealed no significant differences between DeepSport and OptoJump outputs (*p* > 0.05), while Bland–Altman plots indicated no fixed or proportional bias, confirming high agreement between the two systems [7]. These findings suggest that DeepSport can serve as a reliable and valid alternative to laboratory-based force platforms, providing multi-parametric, automated analysis that reduces user-dependent errors compared to traditional jump meters or manual video-based applications such as My Jump [10].

From a practical perspective, DeepSport’s accessibility, automation, and demonstrated accuracy make it a valuable tool for field-based athlete monitoring, enabling coaches and sports scientists to track performance changes, tailor interventions, and implement data-driven decisions without the need for costly laboratory equipment. Nonetheless, limitations should be considered, including the sequential data collection protocol and the exclusive focus on elite basketball players, which may affect generalizability. Future research should examine DeepSport’s applicability across different sports, age groups, and performance levels, as well as its potential integration with real-time feedback and longitudinal monitoring. Overall, these results support DeepSport as a scientifically robust and practically feasible tool that can contribute both methodologically and operationally to sports performance assessment [15,16].

## 5. Conclusions

The AI-based DeepSport application demonstrates excellent reliability and good validity for vertical jump assessment when compared to the OptoJump system. The system’s accessibility, combined with its demonstrated measurement quality, positions it as a valuable tool for sports practitioners seeking field-based athlete assessment solutions. These findings support the integration of AI-based technologies in sports performance assessment, potentially expanding access to sophisticated biomechanical analysis tools.

According to the regression analysis results shown in the figure, the R^2^ values were calculated as follows:

- CMJ (cm): R^2^ = 0.210;
- CMJ Anaerobic Power (W): R^2^ = 0.020;
- SJ (cm): R^2^ = 0.272;
- SJ Anaerobic Power (W): R^2^ = 0.104.

These values support that the measurement results between the devices are randomly distributed without systematic bias. Therefore, it can be stated that the measurements of the DeepSport and OptoJump devices are consistent and reliable. These results underscore the reliability and consistency of the devices in accordance with previous findings in the literature.

Based on the findings of the study, the DeepSport application, when compared to the OptoJump device considered as the reference, demonstrates an excellent level of performance in terms of validity and reliability. An examination of the ICC, CV, SEM, and SDC values indicates that the outcomes produced by the DeepSport application—and by extension both devices—yield valid and reliable results and demonstrate consistency.

No significant difference was detected between the test results of the reference OptoJump and the AI-based DeepSport application. This finding indicates that both devices produce similar results and do not differ systematically.

According to the Bland–Altman analysis, the distribution is random, and no systematic bias is observed. Additionally, the R^2^ values support that neither device exhibits bias.

All results indicate that the AI-based DeepSport application, by yielding outcomes like those of the reference measurement systems, can be considered a valid and reliable alternative. Because it is portable and easily accessible, it can serve as an effective measurement tool in both laboratory and field applications.

These data demonstrate that sports scientists and coaches can reliably employ both devices in the performance analysis of athletes. According to these findings, the NCAA football ranking system is an artificial intelligence model in which precise and clear results are like our study. In addition, it is stated that validity and reliability studies, especially their results, are important and are an important method for basic movement skill assessment tools.

## Figures and Tables

**Table 1:** Comparison of mean jump heights and between DeepSport and OptoJump.

Measurement Type	DeepSport (Mean ± SD)	OptoJump (Mean ± SD)
**CMJ Height (cm)**	**42.3 ± 4.8**	**42.7 ± 4.9**
**SJ Height (cm)**	**38.7 ± 4.2**	**39.1 ± 4.3**

**Table 2:** Test–retest reliability analysis results for measurements obtained with DeepSport and OptoJump devices.

Variables	Device	ICC (95% CI)	CV (%)	SEM	SDC
CMJ (cm)	DeepSport	0.90 (0.674–0.970)	4.86	0.078	0.216
	OptoJump	0.90 (0.680–0.974)	4.36	0.081	0.224
CMJ Anaerobic Power (W)	DeepSport	0.91 (0.712–0.986)	4.95	0.061	0.169
	OptoJump	0.90 (0.693–0.972)	4.79	0.065	0.180
SJ (cm)	DeepSport	0.90 (0.682–0.979)	2.64	0.083	0.230
	OptoJump	0.90 (0.677–0.975)	2.12	0.080	0.221
SJ Anaerobic Power (W)	DeepSport	0.90 (0.708–0.983)	4.60	0.059	0.163
	OptoJump	0.91 (0.710–0.986)	4.27	0.062	0.171

CMJ: CounterMovement Jump; SJ: Squat Jump.

**Table 3:** Paired-samples *t*-test results for measurements obtained with DeepSport and OptoJump devices.

Measurement	Device	*n*	$\bar{X}$	SD	t	*p*
CMJ (cm)	DeepSport	12	40.58	1.975	−0.248	0.809
	OptoJump	12	40.66	1.775		
CMJ Anaerobic Power (W)	DeepSport	12	832.61	91.176	−0.254	0.804
	OptoJump	12	833.43	90.010		
SJ (cm)	DeepSport	12	41.75	1.959	2.057	0.064
	OptoJump	12	40.91	1.083		
SJ Anaerobic Power (W)	DeepSport	12	844.11	89.555	1.924	0.081
	OptoJump	12	835.70	85.877		

CMJ: CounterMovement Jump; SJ: Squat Jump.

## Data Availability

Data are contained within the article.

## Abbreviations

CMJ	Countermovement Jump
SJ	Squat Jump
W	Power
ICC	Intraclass correlation coefficient
SDC	Smallest detectable change
CV	Coefficient of variation
ES	Effect size
SEM	Standard error of measurement
MDC	Minimal detectable change
OLP	Ordinary Least Products regression
